# Complete remission of a high-risk, locally advanced cervical cancer with para-aortic lymph node metastases treated with first-line tislelizumab plus bevacizumab combined with chemotherapy followed by radiotherapy with maintenance therapy: a case report

**DOI:** 10.3389/fimmu.2025.1573202

**Published:** 2025-05-01

**Authors:** Juan Lang, Qianqian Liu, Rong Ji, Miao Qiu, Siben Wang, Qingmeng Liu, Dapeng Li, Ping Chen, Zhongkui Xiong

**Affiliations:** ^1^ Department of Pathology, Shaoxing People’s Hospital, Shaoxing, Zhejiang, China; ^2^ School of Medicine, Shaoxing University, Shaoxing, Zhejiang, China; ^3^ Department of Radiation Oncology, Shaoxing Second Hospital, Shaoxing, Zhejiang, China; ^4^ Department of Pathology, Shaoxing Second Hospital, Shaoxing, Zhejiang, China; ^5^ Department of Radiology, The Second Affiliated Hospital of Nanjing Medical University, Nanjing, Jiangsu, China; ^6^ Department of Obstetrics and Gynecology, Shaoxing Second Hospital, Shaoxing, Zhejiang, China

**Keywords:** cervical cancer, para-aortic lymph node metastases, tislelizumab, bevacizumab, programmed cell death receptor-1, radiochemotherapy

## Abstract

Newly diagnosed cervical cancer with metastatic para-aortic lymph node (PALN) involvement is associated with a significantly poor prognosis, with distant metastasis being the predominant pattern of treatment failure. The programmed cell death receptor-1 (PD-1) pathway has garnered considerable attention due to its role in enabling tumor cells to evade immune surveillance by eliciting the immune checkpoint response of T cells, rendering them highly refractory to conventional chemotherapy. The National Comprehensive Cancer Network (NCCN) guidelines currently recommend pembrolizumab for locally advanced cervical cancer patients positive for PD-L1 (CPS ≥1), as determined by an FDA-approved assay. Tislelizumab, an anti-PD-1 monoclonal IgG4 antibody, has been investigated in hematological malignancies and advanced solid tumors. Nevertheless, literature on regimens incorporating tislelizumab for the treatment of locally advanced cervical cancer is scarce. Herein, we present a case of a newly diagnosed high-risk, locally advanced cervical cancer patient with PALN metastases and low PD-L1 expression, treated with a combination of tislelizumab, bevacizumab, and a platinum-containing chemotherapy regimen followed by radiotherapy with maintenance therapy, resulting in a notable extension of progression-free survival.

## 1 Introduction

Cervical cancer ranks among the most common malignancies worldwide in both incidence and mortality (1). Lymph node status is a critical prognostic factor for survival in early-stage cervical cancer, as outlined by the American Joint Committee on Cancer (AJCC) 8th edition staging criteria (2). A lymph node ratio (LNR) of ≥0.05 has been identified as an independent prognostic factor associated with decreased disease-free survival (DFS) and overall survival (OS) in stage IIIC cervical carcinoma (3). A meta-analysis further supports LNR as an adverse prognostic factor for both OS and DFS in cervical cancer cases (4). Para-aortic lymph node (PALN) involvement has been demonstrated to significantly reduce OS in cervical cancer patients, regardless of the primary tumor size (5). PALN positivity is observed in approximately 17% of cases, representing a small yet particularly challenging subgroup to treat (6). The presence of PALN metastasis is strongly associated with a higher risk of distant recurrence (7). Newly diagnosed cervical cancer with metastatic PALN treated with standard chemoradiation therapy has a notably poor prognosis, with 3-year progression-free survival (PFS) and OS rates of 34% and 39%, respectively (8). Distant metastasis remains the predominant pattern of treatment failure in these cases (9).

In patients with cervical cancer, immune function is often impaired compared to that of healthy women and those with cervical intraepithelial neoplasia (10). Immunotherapy now offers the potential to establish new standards of care in cervical cancer treatment (11). The programmed cell death receptor-1 (PD-1) pathway has gained significant attention for its role in activating the immune checkpoint response of T cells, enabling tumor cells to evade immune surveillance and demonstrating resistance to conventional chemotherapy (12). The NCCN guidelines recommend pembrolizumab for patients whose tumors express PD-L1 (CPS ≥1), as determined by an FDA-approved assay (13).

Tislelizumab, an anti-PD-1 monoclonal IgG4 antibody, has been studied in hematological malignancies and advanced solid tumors (14). Nevertheless, reports on protocols incorporating tislelizumab for the treatment of locally advanced cervical cancer are scarce. Herein, we present a case of a newly diagnosed high-risk, locally advanced cervical cancer patient with PALN metastases and low PD-L1 expression, treated with a combination of tislelizumab, bevacizumab, and chemotherapy, followed by radiotherapy with maintenance therapy.

## 2 Case report

**Figure 1:**
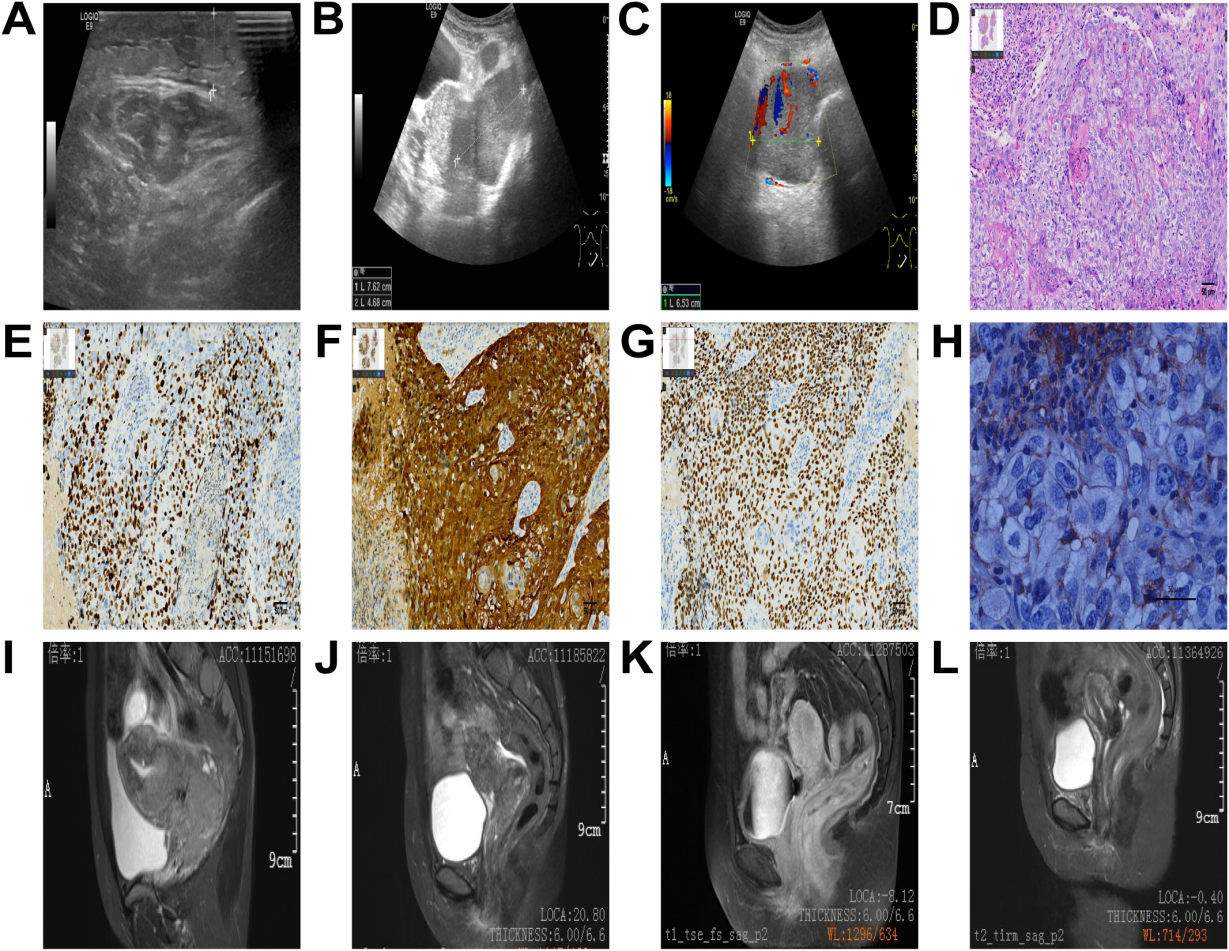
Ultrasound images **(A-C)**. Edema and thickening of the subcutaneous soft tissue in the left lower limb **(A)**, an enlarged cervix **(B)**, and hypoechoic masses in the bilateral adnexal regions **(C)**. Pathological results. Scale bar = 50 μm **(D-H)**. The magnification ratio is 200 times **(D-G)**, or 400 times **(H)**. Medium to poorly differentiated squamous cell carcinoma of the cervix, as demonstrated in the hematoxylin and eosin (H&E) stained image **(D)**. Immunohistochemistry **(E-H)**, Ki67(+70%) **(E)**, P16 (+) **(F)**, P63 (+), P40 (+) **(G)**, PD-L1 TPS (1%-5% +) **(H)**, which was detected using the PD-L1 (28–8) antibody on the DAKO Platform **(H)**. MR images **(I-L)**. Significant cervical enlargement with marked thickening of the posterior wall, blurred signals, and uneven enhancement following contrast administration on day 5 **(I)**, progressively retreated from day 56 **(J)** to day 206 **(K)** until achieving complete remission on day 330 **(L)**.

**Figure 2:**
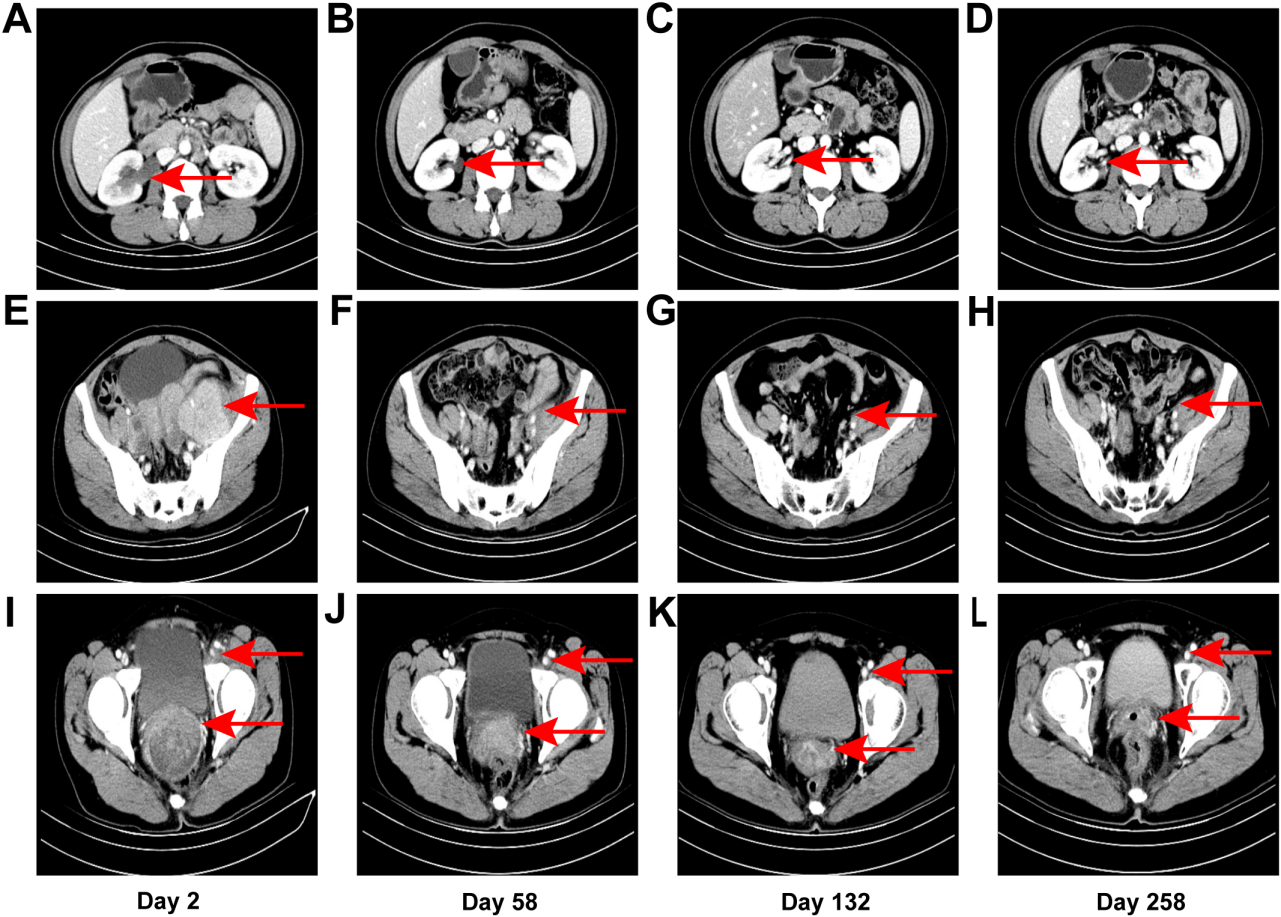
CT images. Right hydronephrosis and right upper ureteral dilation secondary to para-aortic lymph node compression on day 2 from initial contact **(A)**, which was completely relieved on subsequent CT examination [day 58 **(B)**, day 132 **(C)**, day 258 **(D)**]. Uneven enhancement, pelvic lymph node enlargement with partial fusion on day 2 **(E)**, which exhibited a gradual reduction in size from day 58 **(F)** to day 132 **(G)**, as well as day 258 **(H)**. Left inguinal lymph node enlargement as denoted by the aforementioned arrows on day 2 **(I)**, which gradually diminished from day 58 **(J)** to day 132 **(K)**, as well as day 258 **(L)**. Significant thickening of the cervix as indicated by the arrows presented below on day 2 **(I)**, which progressively underwent atrophy from day 58 **(J)** to day 132 **(K)**, as well as day 258 **(L)**.

The patient, a 38-year-old Chinese woman, visited the Department of Gynecology at Shaoxing Second Hospital on May 3, 2022, due to progressive swelling of the left calf for over ten days and newly identified cervical lesions. The patient had a cesarean section 15 years prior and mentioned that her father passed away from leukemia according to her family medical history. She indicated no personal or family occurrences of irregular vaginal bleeding, deep vein thrombosis, or mental health issues. Additionally, she characterized her family relationships as consistent and encouraging. A gynecological examination revealed a nullipara with a smooth vagina and an enlarged cervix that contained a cauliflower-shaped mass approximately 5 cm in diameter. The mass was friable, easily bled, had a hard texture, and involved the pelvic wall. An ultrasound performed on the day before initial contact, showed edema and thickening of the subcutaneous soft tissue in the left lower limb (**Figure 1A**). A subsequent ultrasound on the day after initial contact, revealed an enlarged cervix (**Figure 1B**) with hypoechoic masses in the bilateral adnexal regions (**Figure 1C**). An enhanced abdominal CT scan on day 2 from initial contact(**Figure 2**), demonstrated hydronephrosis and dilation of the right upper ureter (**Figure 2A**), indicative of potential tumor involvement. Additionally, there was evidence of pelvic lymph node enlargement with partial fusion (**Figure 2E**) and para-aortic lymph node enlargement. Notably, a significant thickening of the cervix was observed (**Figure 2I**), accompanied by a cervical mass measuring approximately 84 mm × 66 mm, characterized by uneven enhancement. Another ultrasound on the same day showed right hydronephrosis. A pathological report on day 2 from initial contact, confirmed medium to poorly differentiated squamous cell carcinoma (SCC) of the cervix, with surface bleeding and necrosis associated with infection (**Figure 1D**). Immunohistochemical staining: Ki67(+70%) (**Figure 1E**) P16 (+) (**Figure 1F**), P63(+), CK5/6 (+), P40 (+) (**Figure 1G**), PD-L1(1%-5% +) (**Figure 1H**). MR enhancement (**Figure 1I**) on day 5 from initial contact, indicated significant cervical enlargement with marked thickening of the posterior wall, blurred signals, and uneven enhancement following contrast administration. Multiple large soft tissue masses were observed in the pelvic cavity and left groin, with masses on the left side surrounding blood vessels and forming fused blocks with indistinct margins. It was observed that progressively retreated from day 56 (**Figure 1J**) to day 206 (**Figure 1K**) until achieving complete remission on day 330 (**Figure 1L**).

**Figure 3:**
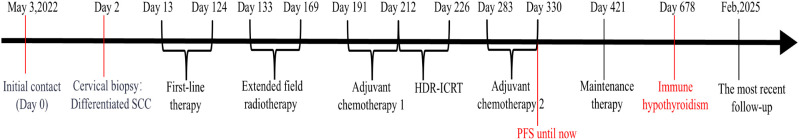
The course timeline illustrating treatments of the patient. The patient underwent 6 cycles of albumin-bound paclitaxel, cisplatin, tislelizumab and bevacizumab shortly after the diagnosis of advanced cervical cancer, followed by extended field radiotherapy (EFRT), 1 cycle of albumin-bound paclitaxel, tislelizumab and bevacizumab, high-dose-rate intracavitary brachytherapy (HDR-ICRT). Subsequently, tislelizumab with or without bevacizumab was administered for maintenance therapy.

On day 2 from initial contact, the patient was diagnosed with cervical cancer, stage IIIC2 (FIGO 2018), with moderately to poorly differentiated squamous carcinoma. After contraindications were excluded, treatment commenced with a regimen of albumin-bound paclitaxel (0.2 g, d1), cisplatin (25 mg, d1–3), tislelizumab (0.2 g, d1), and bevacizumab (0.375 g, d1) administered three weeks per cycle for six cycles from day 13 to day 124 after initial contact. Extended field radiotherapy (EFRT) using 6 MV X-rays followed, with doses of PGTV DT = 5880 cGy/28 Fx and PTV DT = 5040 cGy/28 Fx, delivered from day 133 after initial contact. Subsequently, one cycle of albumin-bound paclitaxel (0.2 g, d1), tislelizumab (0.2 g, d1), and bevacizumab (0.4 g, d1) was administered on day 191. High-dose-rate intracavitary brachytherapy (HDR-ICRT) was performed from day 212 to day 226 from initial contact, with DT = 3000 cGy/5 Fx or 2800 cGy/4 Fx. Further chemotherapy cycles included albumin-bound paclitaxel (0.2 g, d1), cisplatin (25 mg, d1–3), tislelizumab (0.2 g, d1), and bevacizumab (0.375 g, d1) three weeks per for cycle for two cycles, followed by maintenance therapy with tislelizumab (0.2 g, d1) and bevacizumab (0.375 g, d1) every three weeks from day 421 to day 720, and tislelizumab (0.2 g, d1) every three to four weeks from day 741 to now. Efficacy evaluation demonstrated complete remission (CR) following a comprehensive diagnosis conducted collaboratively by an radiation oncologist, a gynecologist, and a radiologist on day 330 from initial contact (**Figure 2L**). During the diagnosis and treatment of patients, changes in the tumor sizes were monitored through CT follow-up examinations. We founded right hydronephrosis and right upper ureteral dilation secondary to para-aortic lymph node compression on day 2 from initial contact (**Figure 2A**), which was completely relieved on subsequent CT examination [day 58 (**Figure 2B**), day 132 (**Figure 2C**), day 258 (**Figure 2D**)]. pelvic lymph node enlargement with partial fusion and uneven enhancement on day 2 (**Figure 2E**), which exhibited a gradual reduction in size from day 58 (**Figure 2F**) to Day 132 (**Figure 2G**) and day 258 (**Figure 2H**). Left inguinal lymph node enlargement as denoted by the aforementioned arrows on day 2 (I), which gradually diminished from day 58 (**Figure 2J**) to day 206 (**Figure 2K**) and day 258 (**Figure 1L**). Significant thickening of the cervix as indicated by the arrows presented below on day 2 (**Figure 2I**), which progressively underwent atrophy from day 58 (**Figure 1J**) to day 206 (**Figure 2K**) and day 258 (**Figure 2L**). Throughout the course of treatment, the following treatment-related adverse reactions were observed: changes in blood routine indicators are presented in **Table 1**. On day 206 from initial contact, aspartate aminotransferase levels were recorded at 38 U/L, which was categorized as a Grade 1 treatment-related adverse reaction based on CTCAE version 4.03. Additionally, on day 678 from the initial contact, the following thyroid function results were obtained: triiodothyronine was 0.56 nmol/l (reference range: 1.25-2.35 nmol/l), total thyroxine was 13.29 nmol/l (reference range: 75–150 nmol/l), free triiodothyronine was 3.33 pmol/l (reference range: 4.00-6.10 pmol/l), free thyroxine was 1.05 pmol/l (reference range: 8.5-14.5 pmol/l), thyroid-stimulating hormone was greater than 50.500 mIU/l (reference range: 0.60-1.90 mIU/l), and thyroid peroxidase antibody was 142.2 IU/ml (reference range: 0-9.0 IU/ml). This abnormality was classified as a Grade 2 treatment-related adverse reaction. In response, levothyroxine therapy was immediately initiated in accordance with the established treatment guidelines. The patient did not experience any adverse events of grade 3 or higher. As of the latest follow-up, the patient’s PFS was 32 months. The course timeline depicting the patient's treatments was illustrated in **Figure 3**.

**Table 1:** The changes in blood routine indicators.

Day	0	13	34	55	75	97	122	146	160	168	188	206	215	222	258	308	330
Hemoglobin, 115-150 g/L	81	75	91	121	122	133	129	117	109	96	106	92	113	123	116	124	128
WBC, 3.5-9.5 × 10^9^/l	9.7	12.9	8.3	5.0	4.8	3.7	4.1	2.5	4.6	3.7	3.6	3.0	2.5	4.9	5.8	4.3	4.0
Granulocyte, 1.8-6.3 × 10^9^/l	8.1	10.5	5.9	2.8	2.5	1.8	2.3	1.6	3.2	3.0	2.4	1.2	3.7	4.6	3.2	3.2	2.8
Platelet, 125-350 × 10^9^/l	528	532	470	347	274	195	185	137	80	101	158	133	188	179	205	215	234

Day, days from initial contact; Red, Below lower limit of the reference interval; Blue, Higher than upper limit of the reference interval.

## 3 Discussion

The patient initially visited the gynecology department at our hospital with two main concerns: gradual enlargement of the left calf over 10 days and newly detected cervical abnormalities. Further investigations, including ultrasound, CT scans, MRI, and cervical core needle biopsy, revealed that the swelling in the left calf was due to enlarged lymph nodes in the left pelvic cavity and groin, rather than lower extremity deep venous thrombosis. Before starting first-line therapy, a multidisciplinary team comprising specialists in gynecology, vascular surgery, and tumor radiotherapy collaborated to devise a comprehensive diagnostic and treatment strategy. This plan considered the patient’s treatment preferences and financial situation. Final approval was obtained only after fully informed consent was given by both the patient and her family. Additionally, through detailed discussions with the patient, we identified three key considerations: treatment efficacy, the risk of adverse events, and survival time with a focus on quality of life.

Following the first treatment cycle, there was significant improvement in the swelling of the left calf. After the second cycle, imaging assessments performed on day 58 showed relief of ureteral obstruction due to regression of pelvic and retroperitoneal lymph nodes, reduction in size of the left inguinal lymph nodes, and marked shrinkage of cervical lesions. Treatment efficacy was classified as partial response (PR). During follow-up, continuous reductions were observed in the sizes of the cervical mass, retroperitoneal lymph nodes, bilateral pelvic lymph nodes, and left inguinal lymph nodes. By day 330 from the initial visit, radiographic evaluations confirmed a CR. Throughout the entire diagnostic and therapeutic process, the patient exhibited excellent tolerance to all treatment modalities. No severe treatment-related safety issues of grade 3 or higher, nor any unexpected adverse events, were noted.

A recent study has shown that the risk factors for cervical cancer include unsafe sexual practices and tobacco use (15). In the AJCC 8th edition, lymph node metastases are not incorporated into the tumor staging system for cervical cancer. However, the revised FIGO 2018 staging for cervical cancer assigns cases with retroperitoneal lymph node involvement to stage IIIC, and those with para-aortic lymph node metastases to stage IIIC2 (16). Cervical cancer patients presenting with tumor sizes exceeding 4 cm, positive lymphovascular space invasion (LVSI), deep cervical stromal invasion, or uterine corpus invasion (UCI) have an increased likelihood of lymph node metastasis (17). While the prognostic significance of pretreatment lymph node metastasis is notable in patients with stage IB1 or IIA cervical cancer, its importance diminishes with advancing disease stages (18). LNR has emerged as a potential prognostic biomarker for predicting disease recurrence in cervical cancer patients treated with radical hysterectomy (19). Metastasized pelvic lymph nodes larger than 1 cm, the presence of multiple pelvic lymph node metastases, common iliac lymph node metastases, and parametrial invasion appear to be independent factors influencing PALN metastases (20). Pelvic lymph node size has been shown to impact OS in patients with advanced cervical cancer (21). Moreover, cervical cancer patients with metastatic lymph nodes greater than 2 cm and SCC histology tend to have poorer survival outcomes compared to those with metastatic lymph nodes measuring 2 cm or less (22). The presence of positive PALN in advanced cervical cancer remains the most significant prognostic factor for survival and plays a critical role in defining treatment strategies (23).

PALN metastasis has been identified as a poor prognostic factor for OS and PFS (24). Patients with positive lymph nodes are likely to benefit more from concurrent chemoradiotherapy (25). Concurrent chemoradiation using platinum-containing chemotherapy is the treatment of choice for cervical cancer patients with stages IB3, II, III, and IVA, as supported by randomized clinical trial results (13). An appropriate and individualized therapeutic strategy, incorporating more intense systemic chemotherapy options in addition to radiotherapy, should be considered based on the presence of PALN metastasis and the patient’s age (7). External beam radiation therapy (EBRT) boost to pelvic lymph nodes has not been shown to reduce recurrence or improve survival in locally advanced cervical cancer with lymph node involvement at diagnosis (26). Overall survival of cervical cancer was significantly influenced by the presence of PALN metastases (27).

The patient with high-risk, locally advanced cervical cancer who underwent EBRT combined with high-dose-rate brachytherapy following first-line treatment. Given the presence of PALN, we adhered to the guideline (28), extended field external beam radiation therapy, also known as extended field external therapy (EFRT), is necessary, encompassing the para-aortic region. The superior boundary should ideally align with the left renal vein; however, it may be required to extend further cephalad to include any involved lymph nodes. Nevertheless, combining concurrent chemotherapy with EFRT seems to provide unclear survival advantages while substantially elevating hematologic toxicity (29). In carefully selected patients with cervical cancer and PALN metastasis, the combination of concurrent chemotherapy with EFRT is linked to improved 5-OS and median OS compared to EFRT alone, while maintaining a tolerable level of acute and late toxicities (30). The survival advantage of combining EFRT with concurrent chemotherapy for cervical cancer with para-aortic lymph node metastasis is still a topic of contention. As a result, the patient in this case received a multidisciplinary approach that combined EFRT, first-line tislelizumab immunotherapy along with bevacizumab and chemotherapy, followed by sequential adjuvant tislelizumab immunotherapy combined with bevacizumab, chemotherapy, and maintenance therapy.

EFRT has proven to be an effective treatment for cervical cancer patients with PALN metastasis (29). A total dose of 50.4 Gy delivered in 1.8 Gy fractions is considered sufficient to control metastatic lymph nodes up to 25 mm in diameter (31). Concurrent chemotherapy with EFRT has been associated with improved 5-year OS and median OS compared to EFRT alone, with a tolerable level of acute and late toxicities in selected patients with cervical cancer and PALN metastases (30). Importantly, this combination treatment has been well tolerated (32). However, there is an opposing view suggesting that the addition of chemotherapy to EFRT may not provide a clear survival benefit and could result in higher hematologic toxicity (29). Furthermore, most patients experienced failure at multiple sites, often outside the treatment field (32). To address the high risk of distant metastases, further improvements in systemic treatment are needed (33). EFRT followed by paclitaxel and carboplatin has been shown to be feasible for women with locally advanced cervical cancer and positive PALN (34).

Despite receiving the recommended treatments, many patients with locally advanced cervical cancer continue to face a poor prognosis (35). With the effective administration of current standards of care, the global community can shift focus to advancing treatment efficacy for these patients (35). Anti-angiogenic therapies, such as bevacizumab, are showing promise in improving outcomes in cervical cancer, as demonstrated in several recent trials (36). The addition of bevacizumab, an anti-angiogenic agent targeting vascular endothelial growth factor 2, to combination chemotherapy in patients with recurrent, persistent, or metastatic cervical cancer was associated with an improvement of 3.7 months in median OS, as shown in the GOG 240 trial (37). Bevacizumab, when combined with chemotherapy, has been shown to increase both OS and PFS, as well as response rates (38). The combination of carboplatin, paclitaxel, and bevacizumab is currently considered the standard frontline treatment for cervical cancer (39). Bevacizumab is expected to remain a key agent in cancer therapy, owing to its established efficacy in approved indications (40).

Studies have shown that PD-1 on tumor-infiltrating lymphocytes (TILs) can bind with programmed death ligand-1 (PD-L1) on tumor cells, which subsequently inhibits T-cell proliferation and activation, leading to immune escape of tumor cells (41). PD-L1 staining was found in 32.2% of 59 cervical cancer samples (42). Chen et al. (41) reported that 64.21% of patients with C-SCC (61/95) expressed PD-L1. Monsrud et al. (43) found PD-L1 positivity in 52 out of 73 (71.2%) cases, with 23 out of 73 (31.5%) showing a CPS ≥10. In a cohort of 118 cervical cancer cases, the PD-L1 positive rate was 58.47%, while microsatellite instability (MSI) status was found in 5.93% of cases (44). PD-L1 positivity was observed in 32 out of 93 (34.4%) cervical carcinomas. Subgroup analyses found that PD-L1 was positive in 28 of 74 (37.8%) squamous cell carcinomas, 2 of 7 (28.6%) adenosquamous carcinomas, and 2 of 12 (16.7%) endocervical adenocarcinomas (45). PD-L1 is universally expressed on the surface of cervical cancer cells. The presence of deficient mismatch repair (dMMR) status has been found to enhance PD-L1 expression in cervical cancer cells. Besides, dMMR status in cervical cancer is associated with the demethylation of the PD-L1 gene promoter region, which may contribute to increased PD-L1 expression (44). In a multi-center retrospective review of 1,093 patients with gynecologic cancers, dMMR or MSI-H was found in 11.3% of cervical cancer cases (46). The pooled prevalence of PD-L1 positivity in cervical cancer patients was 58.1% (47). A retrospective analysis of non-immunotherapy-treated patients with advanced cervical cancer revealed a higher prevalence of PD-L1 CPS ≥1 (48), suggesting that PD-L1 positive patients tend to present at more advanced stages of the disease (43). Furthermore, an increase in PD-L1 gene copy number may serve as a novel prognostic and potential predictive biomarker for anti-PD-1/PD-L1 therapy in locally advanced cervical cancer (49). PD-L1 positive ratios in patients with cervical cancer are shown in **Table 2**.

**Table 2:** PD-L1 positive ratios in patients with cervical cancer.

	Total samples	PD-L1 positivity	Ratio	Histology	Authors
1	59	19	32.2%	–	Grochot RM, et al. (41)
2	95	61	64.21%	squamous cell carcinoma	Chen et al. (40)
3	73	52 (CPS ≥1)	71.2%	squamous cell carcinoma	Monsrud et al. (42)
		23 (CPS≥10)	31.5%		
4	118	69	58.47%	–	Guo F, et al. (43)
5	–	–	58.1%	–	Fu H, et al. (46)
6	93	32	34.4%	–	Reddy OL, et al. (44)
	74	28	37.8%	squamous cell carcinomas	
	7	2	28.6%	adenosquamous carcinomas	
	12	2	16.7%	endocervical adenocarcinomas	
7	143	41 (CPS ≥1)	28.7%	–	Lovane L, et al. (65)

Before 2021, pembrolizumab was the only immunotherapy approved by the United States Food and Drug Administration for cervical cancer, specifically in the second-line setting for recurrent or metastatic (R/M) cases (11). Studies demonstrated that PFS and OS were significantly longer with pembrolizumab compared to placebo among patients with persistent, recurrent, or metastatic cervical cancer who were also receiving chemotherapy, with or without bevacizumab (50). Pembrolizumab combined with chemotherapy, with or without bevacizumab, continued to show clinically meaningful improvements in OS for this patient category (51). Adding pembrolizumab to chemotherapy, with or without bevacizumab, improved OS across various subgroups of patients with persistent, recurrent, or metastatic cervical cancer (52). Pembrolizumab monotherapy also demonstrated durable antitumor activity and a manageable safety profile in patients with advanced cervical cancer (53). Furthermore, the BEATcc trial showed that adding atezolizumab to a standard bevacizumab and platinum-based regimen for metastatic, persistent, or recurrent cervical cancer significantly improved PFS and OS (54). In the innovaTV 204/GOG-3023/ENGOT-cx6 trial, tisotumab vedotin exhibited clinically meaningful and durable antitumor activity with a manageable and tolerable safety profile in women with previously treated recurrent or metastatic cervical cancer (55). In previously treated patients with PD-L1-positive advanced cervical cancer, serplulimab combined with nab-paclitaxel showed durable clinical activity and a manageable safety profile (56). Sintilimab, in combination with anlotinib, proved to be an effective and safe second-line or later treatment option for patients with advanced cervical cancer who had failed prior chemotherapy (57). In addition, balstilimab demonstrated meaningful and durable clinical activity with a manageable safety profile in a phase II clinical trial involving patients with previously treated recurrent or metastatic cervical cancer (58).

The combination of PD-1 inhibitors with concurrent chemoradiotherapy showed potential benefits in terms of tumor response and PFS in patients with locally advanced cervical cancer and pelvic and/or para-aortic lymph node metastases (59). Toripalimab combined with concurrent chemoradiotherapy (CCRT) demonstrated good tolerance and promising anti-tumor effects in patients with locally advanced cervical cancer. Pembrolizumab combined with chemoradiotherapy significantly improved PFS and OS in patients with newly diagnosed, high-risk, locally advanced cervical cancer (60), which provided evidences to support the implementation of this immuno-chemoradiotherapy strategy as a novel standard of care for this patient population (61). In contrast, durvalumab with concurrent chemoradiotherapy did not significantly improve PFS in a biomarker-unselected, all-comers population with locally advanced cervical cancer (62). Nonetheless, concurrent durvalumab plus chemoradiotherapy warrants further exploration in patients with high tumoral PD-L1 expression (62).

The primary metrics for evaluating the efficacy of cancer treatments are PFS and OS. Of these, OS is considered the most reliable indicator; however, data collection for OS is often challenging due to the extended follow-up period required. PFS plays a crucial role in clinical trials and has been formally recognized by both the U.S. Food and Drug Administration (FDA) and the European Medicines Agency (EMA) as an alternative endpoint for assessing treatment efficacy. In certain clinical trials, discrepancies may occur between PFS and OS data, with some drugs demonstrating favorable PFS outcomes but failing to yield significant improvements in OS. According to the EMA Guidelines for the Clinical Evaluation of Anticancer Drugs in Humans (Sixth Edition, 2024), when PFS or DFS is selected as the primary endpoint in a clinical trial, OS should be designated as the secondary endpoint, and vice versa.

Tislelizumab, a modified anti-tumor PD-1 antibody (63), uniquely binds to the CC′ loop of PD-1 with a slow dissociation rate and complete PD-L1 blockage (64). In a retrospective study, tislelizumab demonstrated promising anti-tumor activity and tolerable toxicity in patients with recurrent or metastatic cervical cancer (65). In an open-label phase II study, tislelizumab combined with anlotinib showed therapeutic efficacy and good tolerability in the treatment of advanced cervical cancer. However, immunotherapy with PD-(L)1 inhibitors did not consistently improve survival in patients with locally advanced cervical cancer.

In conclusion, tislelizumab, bevacizumab, and a platinum-containing chemotherapy regimen followed by radiotherapy with maintenance therapy for newly diagnosed, high-risk, locally advanced cervical cancer patients with para-aortic lymph node metastases and PD-L1 expression demonstrated significant clinical efficacy while maintaining an excellent safety profile, with no severe adverse events observed. The patient presented with a high tumor burden, but following multimodal combination therapy, the tumor rapidly decreased in size and maintained both imaging- and clinically-documented tumor-free status. This outcome created favorable conditions for subsequent treatment and potentially improved long-term survival prospects. However, increasing the frequency of testing before and during treatment could aid in selecting more precise and targeted therapies. These may include the following considerations (1): National Comprehensive Cancer Network (NCCN) 2025 v3 guidelines recommend human epidermal growth factor receptor-2 (HER-2) immunohistochemistry (IHC) testing for advanced, metastatic, or recurrent cervical carcinoma, with reflex to HER-2 fluorescence *in situ* hybridization (FISH) if IHC results are inconclusive (2). Testing for mismatch repair (MMR)/microsatellite instability (MSI), tumor mutational burden (TMB), NeuroTrophin Receptor Kinase (NTRK), and proto-oncogene tyrosine-protein kinase receptor (RET) can assist in identifying potential therapeutic targets for patients (3). There are multiple subtypes of HPV, each associated with distinct pathogenic features and prognostic implications. It is advisable to determine HPV status in all cases of cervical adenocarcinoma. HPV *in situ* hybridization (ISH) or molecular testing is preferred; however, p16 immunohistochemistry can serve as an acceptable alternative when HPV-specific testing is unavailable. In this case, the patient was diagnosed with cervical squamous cell carcinoma, and only p16 immunohistochemistry was performed, rather than HPV *in situ* hybridization (ISH) (4). No biopsy was conducted post-cervical treatment to confirm whether a complete pathological response had been achieved (5). Circulating tumor cell testing was not performed at the time of diagnosis or during the course of treatment (6). Lastly, but equally importantly, further investigation in larger clinical cohorts is essential to confirm these results and establish this regimen as a standard of care for locally advanced cervical cancer.

## Data Availability

The original contributions presented in the study are included in the article/supplementary material. Further inquiries can be directed to the corresponding authors.

## References

[1] Gopu P Antony F Cyriac S Karakasis K Oza AM. Updates on systemic therapy for cervical cancer. Indian J Med Res. (2021) 154:293–302. doi: 10.4103/ijmr.IJMR_4454_20 35295013 PMC9131767

[2] Balaya V Guani B Pache B Durand YG Bonsang-Kitzis H Ngo C. Sentinel lymph node in cervical cancer: time to move forward. Chin Clin oncology. (2021) 10:18. doi: 10.21037/cco-21-5 33951917

[3] Aslan K Meydanli MM Oz M Tohma YA Haberal A Ayhan A. The prognostic value of lymph node ratio in stage IIIC cervical cancer patients triaged to primary treatment by radical hysterectomy with systematic pelvic and para-aortic lymphadenectomy. J gynecologic oncology. (2020) 31:e1. doi: 10.3802/jgo.2020.31.e1 PMC6918892 31788991

[4] Cui H Huang Y Wen W Li X Xu D Liu L. Prognostic value of lymph node ratio in cervical cancer: A meta-analysis. Medicine. (2022) 101:e30745. doi: 10.1097/MD.0000000000030745 36281189 PMC9592518

[5] Yang X An J Zhang Y Yang Y Chen S Huang M. Prognostic nomograms predicting survival in patients with locally advanced cervical squamous cell carcinoma: the first nomogram compared with revised FIGO 2018 staging system. Front oncology. (2020) 10:591700. doi: 10.3389/fonc.2020.591700 PMC7606940 33194752

[6] Leblanc E Gauthier H Querleu D Ferron G Zerdoud S Morice P. Accuracy of 18-fluoro-2-deoxy-D-glucose positron emission tomography in the pretherapeutic detection of occult para-aortic node involvement in patients with a locally advanced cervical carcinoma. Ann Surg oncology. (2011) 18:2302–9. doi: 10.1245/s10434-011-1583-9 21347790

[7] Kilic C Kimyon Comert G Cakir C Yuksel D Codal B Kilic F. Recurrence pattern and prognostic factors for survival in cervical cancer with lymph node metastasis. J obstetrics gynaecology Res. (2021) 47:2175–84. doi: 10.1111/jog.14762 33765693

[8] Varia MA Bundy BN Deppe G Mannel R Averette HE Rose PG. Cervical carcinoma metastatic to para-aortic nodes: extended field radiation therapy with concomitant 5-fluorouracil and cisplatin chemotherapy: a Gynecologic Oncology Group study. Int J Radiat oncology biology physics. (1998) 42:1015–23. doi: 10.1016/S0360-3016(98)00267-3 9869224

[9] Liu X Wang W Meng Q Zhang F Hu K. Extended-field intensity-modulated radiation therapy combined with concurrent chemotherapy for cervical cancer with para-aortic lymph nodes metastasis. Japanese J Clin oncology. (2019) 49:263–9. doi: 10.1093/jjco/hyy184 30668725

[10] Zhang L Zhang H Huang Y Xi X Sun Y. Expression of immune cell markers and tumor markers in patients with cervical cancer. Int J gynecological Cancer. (2020) 30:969–74. doi: 10.1136/ijgc-2020-001254 32518078

[11] Monk BJ Enomoto T Kast WM McCormack M Tan DSP Wu X. Integration of immunotherapy into treatment of cervical cancer: Recent data and ongoing trials. Cancer Treat Rev. (2022) 106:102385. doi: 10.1016/j.ctrv.2022.102385 35413489 PMC10697630

[12] Wu X Gu Z Chen Y Chen B Chen W Weng L. Application of PD-1 blockade in cancer immunotherapy. Comput Struct Biotechnol J. (2019) 17:661–74. doi: 10.1016/j.csbj.2019.03.006 PMC6558092 31205619

[13] Abu-Rustum NR Yashar CM Arend R Barber E Bradley K Brooks R. NCCN guidelines(R) insights: cervical cancer, version 1.2024. J Natl Compr Cancer Network: JNCCN. (2023) 21:1224–33. doi: 10.6004/jnccn.2023.0062 38081139

[14] Lee A Keam SJ. Tislelizumab: first approval. Drugs. (2020) 80:617–24. doi: 10.1007/s40265-020-01286-z 32185681

[15] Li T Zhang H Lian M He Q Lv M Zhai L. Global status and attributable risk factors of breast, cervical, ovarian, and uterine cancers from 1990 to 2021. J Hematol oncology. (2025) 18:5. doi: 10.1186/s13045-025-01660-y PMC11721161 39794860

[16] Bhatla N Berek JS Cuello Fredes M Denny LA Grenman S Karunaratne K. Revised FIGO staging for carcinoma of the cervix uteri. Int J gynaecology obstetrics: Off Organ Int Fed Gynaecology Obstetrics. (2019) 145:129–35. doi: 10.1002/ijgo.2019.145.issue-1 30656645

[17] Cao L Kong W Li J Song D Jin B Liu T. Analysis of lymph node metastasis and risk factors in 975 patients with FIGO 2009 stage IA-IIA cervical cancer. Gynecologic obstetric investigation. (2023) 88:30–6. doi: 10.1159/000527712 36450266

[18] Jeong SY Park H Kim MS Kang JH Paik ES Lee YY. Pretreatment lymph node metastasis as a prognostic significance in cervical cancer: comparison between disease status. Cancer Res Treat. (2020) 52:516–23. doi: 10.4143/crt.2019.328 PMC7176965 31671937

[19] Lee YH Chong GO Kim SJ Hwang JH Kim JM Park NJ. Prognostic value of lymph node characteristics in patients with cervical cancer treated with radical hysterectomy. Cancer Manage Res. (2021) 13:8137–45. doi: 10.2147/CMAR.S332612 PMC8560076 34737642

[20] Ayhan A Aslan K Oz M Tohma YA Kuscu E Meydanli MM. Para-aortic lymph node involvement revisited in the light of the revised 2018 FIGO staging system for cervical cancer. Arch gynecology obstetrics. (2019) 300:675–82. doi: 10.1007/s00404-019-05232-7 31263988

[21] Pinto PJJ Chen MJ Santos Neto E Faloppa CC De Brot L Guimaraes APG. Prognostic factors in locally advanced cervical cancer with pelvic lymph node metastasis. Int J gynecological Cancer. (2022) 32:239–45. doi: 10.1136/ijgc-2021-003140 35256409

[22] Aslan K Haberal A Akilli H Meydanli MM Ayhan A. Prognostic value of the number of the metastatic lymph nodes in locally early-stage cervical cancer: squamous cell carcinoma versus non-squamous cell carcinoma. Arch gynecology obstetrics. (2021) 304:1279–89. doi: 10.1007/s00404-021-06030-w 33772630

[23] Gonzalez-Benitez C Salas P Grabowski JP Hernandez A De Santiago J Zapardiel I. Lack of survival benefit of para-aortic lymphadenectomy in advanced cervical cancer. Gynecologic obstetric investigation. (2019) 84:407–11. doi: 10.1159/000497350 30844792

[24] Liu J Tang G Zhou Q Kuang W. Outcomes and prognostic factors in patients with locally advanced cervical cancer treated with concurrent chemoradiotherapy. Radiat oncology. (2022) 17:142. doi: 10.1186/s13014-022-02115-1 PMC9386993 35978412

[25] Ke Y Zhang Z Li Y Qin Y Yang Q Zheng C. Prognostic value of lymph node ratio in patients with non-metastatic cervical cancer treated with radical hysterectomy: A population-based study. Eur J Surg oncology: J Eur Soc Surg Oncol Br Assoc Surg Oncology. (2024) 50:108258. doi: 10.1016/j.ejso.2024.108258 38484490

[26] Wujanto C Choo BA Tan D Ilancheran A Ng J Low JJH. Does external beam radiation boost to pelvic lymph nodes improve outcomes in patients with locally advanced cervical cancer? BMC Cancer. (2019) 19:385. doi: 10.1186/s12885-019-5594-4 31023261 PMC6485109

[27] Vandeperre A Van Limbergen E Leunen K Moerman P Amant F Vergote I. Para-aortic lymph node metastases in locally advanced cervical cancer: Comparison between surgical staging and imaging. Gynecologic oncology. (2015) 138:299–303. doi: 10.1016/j.ygyno.2015.05.021 26007204

[28] Keenan LG Rock K Azmi A Salib O Gillham C McArdle O. An atlas to aid delineation of para-aortic lymph node region in cervical cancer: Design and validation of contouring guidelines. Radiotherapy oncology: J Eur Soc Ther Radiol Oncology. (2018) 127:417–22. doi: 10.1016/j.radonc.2018.02.013 29523410

[29] Yoon HI Cha J Keum KC Lee HY Nam EJ Kim SW. Treatment outcomes of extended-field radiation therapy and the effect of concurrent chemotherapy on uterine cervical cancer with para-aortic lymph node metastasis. Radiat oncology. (2015) 10:18. doi: 10.1186/s13014-014-0320-5 PMC4311470 25582425

[30] Ng BH Rozita A Adlinda A Lee WC Wan Zamaniah W. Extended field radiotherapy with or without chemotherapy in patients with cervical cancer and positive para-aortic lymph nodes: a single institution retrospective review. Asian Pacific J Cancer prevention: APJCP. (2015) 16:3827–33. doi: 10.7314/APJCP.2015.16.9.3827 25987044

[31] Hata M Miyagi E Koike I Numazaki R Asai-Sato M Kasuya T. Radiation therapy for para-aortic lymph node metastasis from uterine cervical cancer. Anticancer Res. (2015) 35:4849–54. 26254377

[32] Rajasooriyar C Van Dyk S Bernshaw D Kondalsamy-Chennakesavan S Barkati M Narayan K. Patterns of failure and treatment-related toxicity in advanced cervical cancer patients treated using extended field radiotherapy with curative intent. Int J Radiat oncology biology physics. (2011) 80:422–8. doi: 10.1016/j.ijrobp.2010.02.026 20494528

[33] Marnitz S Schram J Budach V Sackerer I Vercellino GF Sehouli J. Extended field chemoradiation for cervical cancer patients with histologically proven para-aortic lymph node metastases after laparaoscopic lymphadenectomy. Strahlentherapie und Onkologie: Organ der Deutschen Rontgengesellschaft [et al]. (2015) 191:421–8. doi: 10.1007/s00066-014-0785-z 25413986

[34] Boardman CH Brady WE Dizon DS Kunos CA Moore KN Zanotti KM. A phase I evaluation of extended field radiation therapy with concomitant cisplatin chemotherapy followed by paclitaxel and carboplatin chemotherapy in women with cervical carcinoma metastatic to the para-aortic lymph nodes: an NRG oncology/gynecologic oncology group study. Gynecologic oncology. (2018) 151:202–7. doi: 10.1016/j.ygyno.2018.08.006 PMC6291247 30174176

[35] Mayadev JS Ke G Mahantshetty U Pereira MD Tarnawski R Toita T. Global challenges of radiotherapy for the treatment of locally advanced cervical cancer. Int J gynecological Cancer. (2022) 32:436–45. doi: 10.1136/ijgc-2021-003001 PMC8921593 35256434

[36] Fisher CM Schefter TE. Profile of bevacizumab and its potential in the treatment of cervical cancer. OncoTargets Ther. (2015) 8:3425–31. doi: 10.2147/OTT.S73251 PMC4657807 26640382

[37] Tewari KS Sill MW Long HJ 3rd Penson RT Huang H Ramondetta LM. Improved survival with bevacizumab in advanced cervical cancer. New Engl J medicine. (2014) 370:734–43. doi: 10.1056/NEJMoa1309748 PMC4010094 24552320

[38] Enriquez-Aceves I Galicia-Carmona T Coronel-Martinez JA Espinosa-Romero R Calderillo-Ruiz G Cortes-Esteban P. Standard treatment with bevacizumab as targeted therapy in cervical cancer. Rev investigacion clinica; organo del Hosp Enfermedades la Nutricion. (2020) 72:213–218. doi: 10.24875/RIC.20000061 33064702

[39] Marquina G Manzano A Casado A. Targeted agents in cervical cancer: beyond bevacizumab. Curr Oncol reports. (2018) 20:40. doi: 10.1007/s11912-018-0680-3 29611060

[40] Garcia J Hurwitz HI Sandler AB Miles D Coleman RL Deurloo R. Bevacizumab (Avastin(R)) in cancer treatment: A review of 15 years of clinical experience and future outlook. Cancer Treat Rev. (2020) 86:102017. doi: 10.1016/j.ctrv.2020.102017 32335505

[41] Chen J Gu P Wu H. Uncovering PD-L1 and CD8(+) TILS expression and clinical implication in cervical squamous cell carcinoma. BioMed Res Int. (2020) 2020:8164365. doi: 10.1155/2020/8164365 32884946 PMC7455844

[42] Grochot RM Brollo J Neto FR Tregnago AC Scholze C Norris R. Expression of PD-L1 in cervical carcinoma and its impact on survival associated with T-cell infiltration and FoxP3 expression. Cancer Manage Res. (2019) 11:4597–605. doi: 10.2147/CMAR.S194597 PMC6529624 31191020

[43] Monsrud AL Avadhani V Mosunjac MB Flowers L Krishnamurti U. Programmed death ligand-1 (PD-L1) expression in cervical squamous cell carcinoma: does it correlate with outcomes? Int J gynecological Pathol. (2023) 42:535–43. doi: 10.1097/PGP.0000000000000975 37562018

[44] Guo F Lu R Kong W Anwar M Feng Y. DNA mismatch repair system regulates the expression of PD-L1 through DNMTs in cervical cancer. Cancer Cell Int. (2024) 24:25. doi: 10.1186/s12935-024-03214-7 38200495 PMC10782574

[45] Reddy OL Shintaku PI Moatamed NA. Programmed death-ligand 1 (PD-L1) is expressed in a significant number of the uterine cervical carcinomas. Diagn pathology. (2017) 12:45. doi: 10.1186/s13000-017-0631-6 PMC5473984 28623908

[46] Noh JJ Kim MK Choi MC Lee JW Park H Jung SG. Frequency of mismatch repair deficiency/high microsatellite instability and its role as a predictive biomarker of response to immune checkpoint inhibitors in gynecologic cancers. Cancer Res Treat. (2022) 54:1200–8. doi: 10.4143/crt.2021.828 PMC9582475 34902958

[47] Fu H Fu Z Mao M Si L Bai J Wang Q. Prevalence and prognostic role of PD-L1 in patients with gynecological cancers: A systematic review and meta-analysis. Crit Rev oncology/hematology. (2023) 189:104084. doi: 10.1016/j.critrevonc.2023.104084 37536446

[48] Baek MH Chen L Tekin C Cristescu R Jin XY Shao C. Prevalence and prognostic value of PD-L1 expression and tumor mutational burden in persistent, recurrent, or metastatic cervical cancer. J gynecologic Oncol. (2024) 35:e105. doi: 10.3802/jgo.2024.35.e105 PMC11543264 38857910

[49] Loharamtaweethong K Supakatitham C Vinyuvat S Puripat N Tanvanich S Sitthivilai U. Prognostic significance of PD-L1 protein expression and copy number gains in locally advanced cervical cancer. Asian Pacific J Allergy Immunol. (2021) 39:309–18. doi: 10.12932/AP-120419-0538 31586491

[50] Colombo N Dubot C Lorusso D Caceres MV Hasegawa K Shapira-Frommer R. Pembrolizumab for persistent, recurrent, or metastatic cervical cancer. New Engl J medicine. (2021) 385:1856–67. doi: 10.1056/NEJMoa2112435 34534429

[51] Monk BJ Colombo N Tewari KS Dubot C Caceres MV Hasegawa K. First-line pembrolizumab + Chemotherapy versus placebo + Chemotherapy for persistent, recurrent, or metastatic cervical cancer: final overall survival results of KEYNOTE-826. J Clin Oncol. (2023) 41:5505–11. doi: 10.1200/JCO.23.00914 37910822

[52] Tewari KS Colombo N Monk BJ Dubot C Caceres MV Hasegawa K. Pembrolizumab or placebo plus chemotherapy with or without bevacizumab for persistent, recurrent, or metastatic cervical cancer: subgroup analyses from the KEYNOTE-826 randomized clinical trial. JAMA oncology. (2024) 10:185–92. doi: 10.1001/jamaoncol.2023.5410 PMC10722390 38095881

[53] Chung HC Ros W Delord JP Perets R Italiano A Shapira-Frommer R. Efficacy and safety of pembrolizumab in previously treated advanced cervical cancer: results from the phase II KEYNOTE-158 study. J Clin Oncol. (2019) 37:1470–8. doi: 10.1200/JCO.18.01265 30943124

[54] Oaknin A Gladieff L Martinez-Garcia J Villacampa G Takekuma M De Giorgi U. Atezolizumab plus bevacizumab and chemotherapy for metastatic, persistent, or recurrent cervical cancer (BEATcc): a randomised, open-label, phase 3 trial. Lancet. (2024) 403:31–43. doi: 10.1016/S0140-6736(23)02405-4 38048793

[55] Coleman RL Lorusso D Gennigens C Gonzalez-Martin A Randall L Cibula D. Efficacy and safety of tisotumab vedotin in previously treated recurrent or metastatic cervical cancer (innovaTV 204/GOG-3023/ENGOT-cx6): a multicentre, open-label, single-arm, phase 2 study. Lancet Oncology. (2021) 22:609–19. doi: 10.1016/S1470-2045(21)00056-5 33845034

[56] An J Li X Wang J Zhu L An R Jiang K. Efficacy and safety of serplulimab plus nab-paclitaxel in previously treated patients with PD-L1-positive advanced cervical cancer: a phase II, single-arm study. Front Immunol. (2023) 14:1142256. doi: 10.3389/fimmu.2023.1142256 37153587 PMC10161140

[57] Xu Q Wang J Sun Y Lin Y Liu J Zhuo Y. Efficacy and safety of sintilimab plus anlotinib for PD-L1-positive recurrent or metastatic cervical cancer: A multicenter, single-arm, prospective phase II trial. J Clin Oncol. (2022) 40:1795–805. doi: 10.1200/JCO.21.02091 PMC9148684 35192397

[58] O'Malley DM Oaknin A Monk BJ Selle F Rojas C Gladieff L. Phase II study of the safety and efficacy of the anti-PD-1 antibody balstilimab in patients with recurrent and/or metastatic cervical cancer. Gynecologic oncology. (2021) 163:274–80. doi: 10.1016/j.ygyno.2021.08.018 34452745

[59] Liu C Ran X Wang Z Zhang K. Efficacy and safety of PD-1 inhibitor combined with concurrent chemoradiotherapy in locally advanced cervical cancer with pelvic and/or para-aortic lymph node metastases: a retrospective cohort study. Chin Clin oncology. (2023) 12:38. doi: 10.21037/cco-23-70 37699603

[60] Lorusso D Xiang Y Hasegawa K Scambia G Leiva M Ramos-Elias P. Pembrolizumab or placebo with chemoradiotherapy followed by pembrolizumab or placebo for newly diagnosed, high-risk, locally advanced cervical cancer (ENGOT-cx11/GOG-3047/KEYNOTE-A18): a randomised, double-blind, phase 3 clinical trial. Lancet. (2024) 403:1341–50. doi: 10.1016/S0140-6736(24)00317-9 38521086

[61] Lorusso D Xiang Y Hasegawa K Scambia G Leiva M Ramos-Elias P. Pembrolizumab or placebo with chemoradiotherapy followed by pembrolizumab or placebo for newly diagnosed, high-risk, locally advanced cervical cancer (ENGOT-cx11/GOG-3047/KEYNOTE-A18): overall survival results from a randomised, double-blind, placebo-controlled, phase 3 trial. Lancet. (2024) 404:1321–32. doi: 10.1016/S0140-6736(24)01808-7 39288779

[62] Monk BJ Toita T Wu X Vazquez Limon JC Tarnawski R Mandai M. Durvalumab versus placebo with chemoradiotherapy for locally advanced cervical cancer (CALLA): a randomised, double-blind, phase 3 trial. Lancet Oncology. (2023) 24:1334–48. doi: 10.1016/S1470-2045(23)00479-5 38039991

[63] Zhang L Geng Z Hao B Geng Q. Tislelizumab: A modified anti-tumor programmed death receptor 1 antibody. Cancer control: J Moffitt Cancer Center. (2022) 29:10732748221111296. doi: 10.1177/10732748221111296 PMC9358212 35926155

[64] Hong Y Feng Y Sun H Zhang B Wu H Zhu Q. Tislelizumab uniquely binds to the CC' loop of PD-1 with slow-dissociated rate and complete PD-L1 blockage. FEBS Open bio. (2021) 11:782–92. doi: 10.1002/2211-5463.13102 PMC7931243 33527708

[65] Zheng X Gu H Cao X Pan B Xiang H Ju M. Tislelizumab for cervical cancer: A retrospective study and analysis of correlative blood biomarkers. Front Immunol. (2023) 14:1113369. doi: 10.3389/fimmu.2023.1113369 36875089 PMC9975598

